# *Shigella* spp. Antimicrobial Drug Resistance, Papua New Guinea, 2000–2009

**DOI:** 10.3201/eid1611.101025

**Published:** 2010-11

**Authors:** Alexander Rosewell, Berry Ropa, Enoch Posanai, Samir R. Dutta, Glen Mola, Anthony Zwi, C. Raina MacIntyre

**Affiliations:** Author affiliations: World Health Organization, Port Moresby, Papua New Guinea (A. Rosewell);; National Department of Health, Port Moresby (B. Ropa, E. Posanai);; Port Moresby General Hospital, Port Moresby (S.R. Dutta);; University of Papua New Guinea, Port Moresby (G. Mola);; University of New South Wales, Sydney, New South Wales, Australia (A. Rosewell, A. Zwi, C.R. MacIntyre)

**Keywords:** Antimicrobial drug resistance, Shigella, resistance, surveillance, bacteria, letter

**To the Editor:** Approximately half the *Shigella* spp. infections in developing countries are caused by endemic shigellae (*1*)*,* which in these countries are responsible for ≈10% of all episodes of diarrhea among children <5 years of age and up to 75% of deaths from diarrhea (*2*). Deaths from epidemic *Shigella* spp. in the community are estimated to outnumber deaths within the healthcare setting. In Papua New Guinea, diarrhea is a major cause of hospital admission and death (*3*); *Shigella* spp. are among the most common causes of enteric bacterial infection (*4**,**5*)*,* and *S. flexneri* is the most common serotype (*3**,**6*)*.* Outbreaks of bloody diarrhea are frequently reported; however, diagnosis in remote settings is challenging, partly because the storage requirements for the organism are difficult to meet.

Multidrug resistance of shigellae is not new (*1*); many countries have reported resistance to amoxicillin, co-trimoxazole, and chloramphenicol. For this reason, the World Health Organization recommends that all patients with bloody diarrhea be treated with either ciprofloxacin or 1 of the 3 second-line drugs: pivmecillinam, azithromycin, and ceftriaxone (*7*)*.* The antimicrobial drug currently recommended for patients with bloody diarrhea in primary healthcare settings in Papua New Guinea is co-trimoxazole (*8*); ciprofloxacin is available only in hospitals.

In August 2009, an epidemic of multidrug-resistant *S. flexneri* infection associated with widespread illness and death across 4 provinces of Papua New Guinea was reported to health authorities. To understand the trends and to inform antimicrobial drug policy makers, we reviewed retrospective microbiological data for 2000–2009. With the exception of 3 isolates collected during an outbreak in the border regions of the 4 provinces during 2009 (excluded from analysis), all isolates in our study were obtained as part of routine surveillance. Fecal samples were collected by clinicians from any patient seeking care for severe diarrhea at Port Moresby General Hospital.

Before serologic testing was conducted, samples were spread directly on desoxycholate citrate agar and MacConkey agar plates for culture. Antimicrobial drug resistance testing was performed by using the Kirby-Bauer method.

From a total of 3,419 fecal samples cultured, 136 (4.0%) were positive for *Shigella* spp. The most commonly isolated species was *S. flexneri* (90.4%); less frequently isolated were *S. boydii* (3.7 %), *S. dysenteriae* (2.9%), and *S. sonnei* (1.5%). Of the 123 *S. flexneri* isolates, 20 (16%) were further characterized; the most frequent serovars were serovar 2 (40%) and serovar 3 (30%). Many (48%) *Shigella* spp.–positive isolates were from children <5 years of age. The highest rates of antimicrobial drug resistance of all *Shigella* spp. were to amoxicillin (96%), co-trimoxazole (86%), and chloramphenicol (60%); no resistance to ciprofloxacin and cephalexin was found (Table).

**Table:** Antimicrobial drug resistance of *Shigella* spp., Papua New Guinea, 2000–2009*

Drug	Total no. isolates tested	Sensitivity	*Shigella* sp., no. (%) isolates					
			*S. boydi*	*S. dysenteriae*	*S. flexneri*	*S. sonneii*	Unknown sp.	Total
Amoxicillin	98	S	0	1 (33)	2 (2)	0	1 (100)	4 (4)
		R	3 (100)	2 (67)	87 (98)	2 (100)	0	94 (96)
Cephalexin	46	S	2 (67)	2 (100)	38 (100)	2 (100)	1 (100)	45 (98)
		I	1 (33)	0	0	0	0	1 (2)
		R	0	0	0	0	0	0
Ciprofloxacin	41	S	2 (67)	NA	35 (100)	1 (100)	2 (100)	40 (98)
		I	1 (33)	NA	0	0	0	1 (2)
		R	0	0	0	0	0	0
Chloramphenicol	114	S	0	2 (50)	9 (9)	2 (100)	1 (50)	14 (12)
		I	0	2 (50)	28 (28)	0	1 (50)	31 (27)
		R	4 (100)	0	64 (63)	0	0	68 (60)
Naladixic acid	13	S	1 (100)	0	8 (100)	1 (100)	1 (50)	11 (85)
		R	0	1 (100)	0	0	1 (50)	2 (15)
Co-trimoxazole	76	S	1 (25)	1 (33)	9 (14)	0	0	11 (14)
		R	3 (75)	2 (67)	57 (86)	2 (100)	1 (100)	65 (86)

*S, sensitive; R, resistant, I, intermediate; NA, not applicable.

Current evidence supports the use of ciprofloxacin, ceftriaxone, and pivmecillinam for treatment of bloody diarrhea (*9*)*.* It also suggests that dysentery rarely relapses if an infected child has received a full course of treatment with 1 of these drugs and the causative pathogen is sensitive to the drug. Reducing the risk for relapse of bacterial infections among children is beneficial because it reduces the likelihood of subsequent episodes of dysentery occurring in that child and of transmission to others (*9*)*.* In our study, most isolates were resistant to co-trimoxazole and the other available antimicrobial drugs, indicating that their use would not have reduced illness and subsequent transmission in this setting. The lack of resistance to ciprofloxacin and cephalexin indicates that these drugs may be more effective; however, they are neither available at the primary healthcare level nor recommended in Papua New Guinea, which is cause for concern.

Surveillance for antimicrobial drug resistance is essential for the containment of antimicrobial drug resistance globally. However, international surveillance depends on strong national surveillance systems. Despite the existence of a network of subnational laboratories where fecal sample cultures had been performed, these laboratories no longer perform these cultures. In 1964, the laboratory in 1 provincial hospital analyzed and subtyped 1,000 stool samples over a 15-month period (*6*)*.* In our study, conducted at the national referral hospital (which limits the representativeness), we analyzed 3,419 fecal samples over a 10-year period.

Outbreaks of bloody diarrhea are common in remote settings in Papua New Guinea, yet with the exception of the 3 isolates from 2009 that were excluded from analysis, no *Shigella* spp.–positive samples have been identified during outbreaks. Molecular methods may serve as an adjunct to traditional laboratory methods by improving sensitivity and also enabling diagnosis of *Shigella* spp. outbreaks among remote populations where specimen storage and transport requirements may be challenging (*10*)*.*

We describe extremely high rates of resistance of *Shigella* spp. to co-trimoxazole, the recommended treatment for bloody diarrhea in Papua New Guinea. Strengthening national surveillance for antimicrobial drug resistance would provide the evidence to better inform policy decision makers. A review of the national antimicrobial drug policy for management of bloody diarrhea is urgently needed.
